# Timing to be precise? An overview of spike timing-dependent plasticity, brain rhythmicity, and glial cells interplay within neuronal circuits

**DOI:** 10.1038/s41380-023-02027-w

**Published:** 2023-03-29

**Authors:** Yuniesky Andrade-Talavera, André Fisahn, Antonio Rodríguez-Moreno

**Affiliations:** 1 Laboratory of Cellular Neuroscience and Plasticity, Department of Physiology, Anatomy and Cell Biology, Universidad Pablo de Olavide, ES-41013 Seville, Spain; 2 Department of Biosciences and Nutrition and Department of Women’s and Children’s Health, Karolinska Institutet, 171 77 Stockholm, Sweden

**Keywords:** Physiology, Neuroscience, Cell biology

## Abstract

In the mammalian brain information processing and storage rely on the complex coding and decoding events performed by neuronal networks. These actions are based on the computational ability of neurons and their functional engagement in neuronal assemblies where precise timing of action potential firing is crucial. Neuronal circuits manage a myriad of spatially and temporally overlapping inputs to compute specific outputs that are proposed to underly memory traces formation, sensory perception, and cognitive behaviors. Spike-timing-dependent plasticity (STDP) and electrical brain rhythms are suggested to underlie such functions while the physiological evidence of assembly structures and mechanisms driving both processes continues to be scarce. Here, we review foundational and current evidence on timing precision and cooperative neuronal electrical activity driving STDP and brain rhythms, their interactions, and the emerging role of glial cells in such processes. We also provide an overview of their cognitive correlates and discuss current limitations and controversies, future perspectives on experimental approaches, and their application in humans.

## Introduction

How do brain circuits manage a plethora of highly diverse inputs to generate precise outputs without functional failure? Neurons—widely regarded as the main functional units of the brain—perform crucial computational tasks by non-linear integration of synaptic inputs and subsequent generation of electrical action potentials (spikes). This computation is governed by multiple factors, including neuronal passive membrane properties, geometry, and active currents [1, 2]. However, these single-neuron properties do not in themselves explain the emergence of complex cognitive processes such as memory recall, planning, and decision-making. In this regard, multiple neurons performing independent computations solely based on their neuronal properties could be likened to members of an orchestra playing temporally uncorrelated melodies: the result will be noise and lack of information content or it may provide certain information that will appear more difficult for the listener to understand.

A plausible explanation for the brain’s operational capacity emerges from the formation and dissolution of neuronal ensembles supported by timely coincident and mainly cooperative activity across activity-synchronized brain circuits [3, 4]. Active neuronal ensembles, also known as neuronal assemblies [3], can be transiently formed and segregated based on synaptic connections’ recruitment, strength, and excitability in a specified discrete circuitry bearing diverse temporal evolutions. One factor supporting the neuronal ability to transiently cooperate and/or engage in a coordinated activity like a “group of discrete units” is their intrinsic resonance. As such, neuronal resonance is supported by diverse ionic mechanisms availing the neurons to selectively respond to inputs of preferred frequencies [5] and can vary within neuronal compartments (e.g., from dendrites to soma) due to the spatial gradients of the density of specific ion channels [6]. Experimentally, an intrinsic difficulty is the determination of assembly sizes since it is not possible to quantitatively define which neurons are bound into the primary assembly at any given time point and which represent feedback activation of assembly members or newly recruited assemblies serving other goals [3]. Therefore, these facts provide a more complex picture for the study of the neuronal circuit’s dynamics that underlie or correlate with higher-order functions.

Therefore, it is assumed that in the mammalian neocortex, information processing relies on the timing precision of neuronal activity within neuronal circuits and oscillators, [7–17] although firing rate is supposed to hold relevance. In this regard, the intrinsic timing of neuronal spiking seemingly depends on balanced subthreshold ionic currents (inward and outward) that shape the excitatory postsynaptic potentials (EPSP). The shape of the EPSP together with the resting membrane potential and firing threshold determine the efficacy of the EPSP-spike coupling during the EPSP-induced postsynaptic firing [18]. This coupling is crucial for the operation of neuronal networks and its timing precision varies across diverse brain areas and neuronal types [18]. In addition, spike timing precision is controlled by the afterhyperpolarization (AHP) that follows the action potentials in a variety of neurons, [19–21] and enhancement of AHP by (e.g.,) persistent Na^+^ current decreases spike time precision in response to single EPSPs [22]. On the contrary, a persistent reduction of the AHP potentiates EPSP-spike coupling, and thus the fidelity of spike timing [23]. Moreover, diverse studies indicate that spike timing is also plastic and that such plasticity and timing precision are highly influenced by ion channels [6, 23–25]. Interestingly, evidence of a role for myelination and consequently oligodendrocytes aiding spike timing in neuronal circuits is recently emerging [26].

**Fig. 1:**
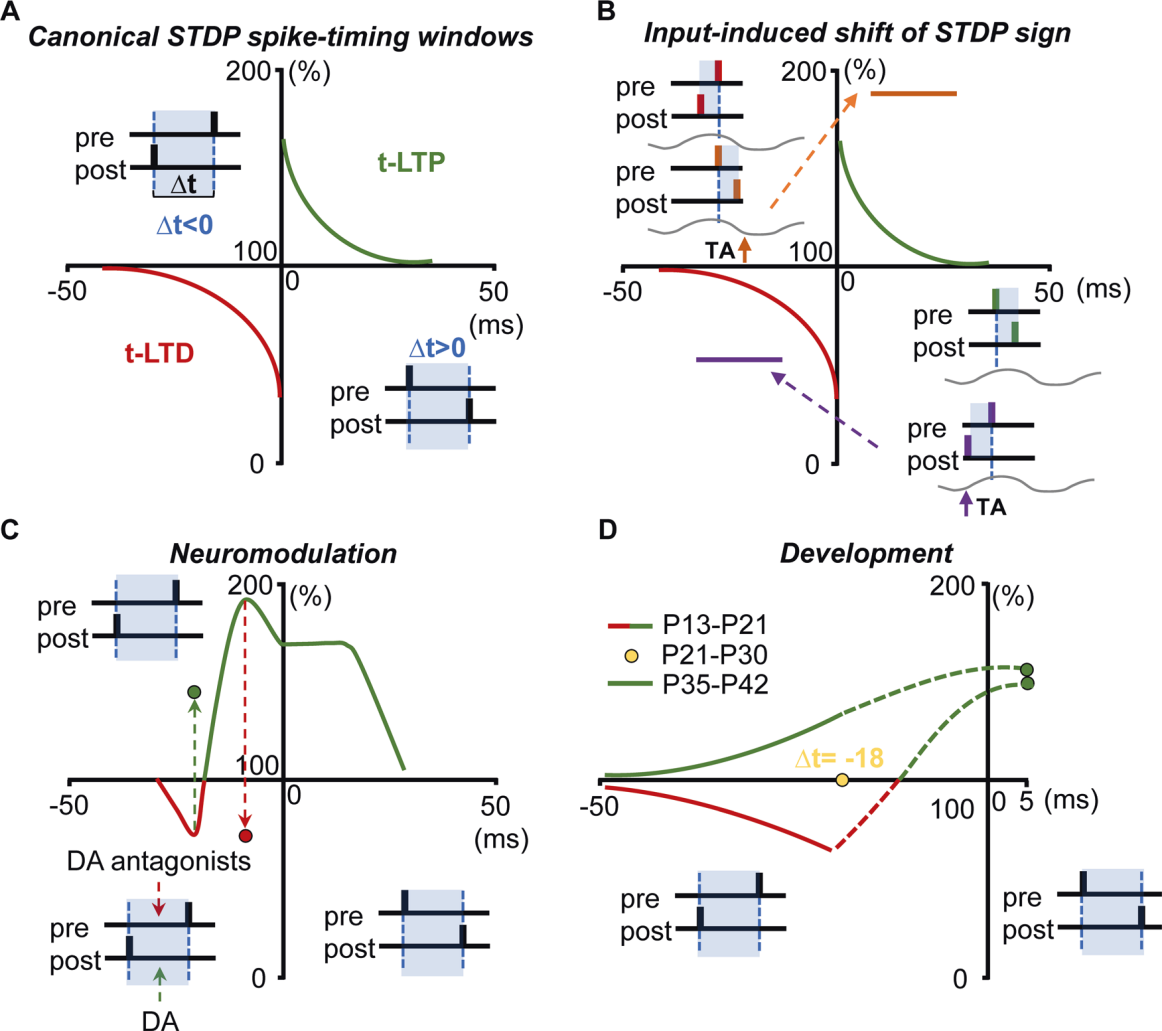
Schematic diagrams showing the diversity of STDP forms and some of the factors that might influence its expression, sign, and magnitude. The relative magnitude (*y*-axis, in %) and spike-time intervals (temporal windows, *x*-axis, in ms) are simplified and presented schematically and arbitrary in **A**–**C**. **A** In the classical concept, STDP is equally balanced for t-LTD and t-LTP: postsynaptic spiking occurring before presynaptic activity drives t-LTD (in red, pre-post timing interval: ∆*t* < 0) whereas presynaptic spiking occurring before postsynaptic spike leads to t-LTP (in green, pre-post timing interval: ∆*t* > 0) [8, 81]. **B** Schematic showing that the sign of STDP is under the control of external input at CA3-CA1 synapses from 13–18 postnatal days (P13–P18) mice [38]. Oscillatory activity (in gray) is induced in a single CA1 pyramidal neuron designed to make the cell firing just one action potential near the peak of the oscillation and temperoammonic input (TA) to CA1 changes the order of correlated pre- and postsynaptic activity relative to ongoing theta wave. Upper left panel: without TA stimulation, post-pre activity coincidence near the theta oscillation peak drives t-LTD (in red) whereas TA stimulation in the descending theta phase (orange arrow) delays the postsynaptic spike changing the order to pre-post coincidence thus driving t-LTP (in dark orange). The spike-time interval for t-LTP and the magnitude (dark orange line) are arbitrary. Bottom right panel: without TA stimulation, pre-post activity coincidence near the theta oscillation peak drives t-LTP (in green) whereas TA stimulation in the ascending theta phase (magenta arrow) advances the postsynaptic spike setting the order to post-pre coincidence that drives t-LTD (in magenta). The spike-timing interval and the magnitude for t-LTD (magenta line) are arbitrary. **C** Dopamine (DA) influences the sign and the time window for STDP in CA3-CA1 synapses at P13–P18. A post-pre pairing protocol with ∆*t* = −20 ms induces t-LTD (in red) while in the presence of DA the same protocol induces t-LTP (green dot). In turn, a post-pre protocol with ∆*t* > −10 ms induces t-LTP (in green). DA antagonism drives t-LTD with a post-pre protocol (red dot) that at ∆*t* = −10 ms induces t-LTP when endogenous DA signaling is intact. This indicates that DA widens the time window for t-LTP which putatively could have an impact on reward-related learning [89, 194]. **D** STDP time window in CA3-CA1 synapses changes during development. A post-pre protocol (∆*t* from −35 to −18 ms) induces t-LTD at P13–P21 (in red). This form of t-LTD requires endocannabinoid signaling and D-serine from astrocytes [76]. At P21–P30, a post-pre pairing (∆*t* = −18 ms) fails to induce t-LTD (yellow dot). The developmental loss of t-LTD at this age involves adenosine type 1 receptors (A_1_Rs) and adenosine released by astrocytes in a calcium-dependent manner [68]. Interestingly, at P35–P42 (in green) post-pre protocol (∆*t* from −35 to −18 ms) induces t-LTP instead of t-LTD. This developmental switch in the plasticity rule for STDP involves the release of adenosine and glutamate by astrocytes indicating that they are key players in the control of temporal windows for STDP during the development of CA3-CA1 synapses. In addition, a pre-post pairing (∆*t* = +5 ms) induces t-LTP at both postnatal ages in this hippocampal synapse [35, 76]. Dashed lines represent arbitrary magnitudes that may follow this behavior according to the findings in [35].

Irrespective of the complex mechanisms underlying timing precision, it is known that coincident firing (in a window ranging from 10 ms to ~100 ms) leads to long-lasting changes in synaptic efficacy: namely spike timing-dependent plasticity (STDP) [8, 27]. STDP has been related to key functional consequences such as circuit refinement and cortical map plasticity [27–30] which could influence learning and memory. In addition, diverse forms have been described [8, 10, 31, 32]. Likewise, in hippocampal CA3-CA1 synapses, STDP has been canonically defined as a bi-directional form of synaptic plasticity that depends on the order and timing of spike occurrence: presynaptic spiking leading postsynaptic spiking drives long-term potentiation (t-LTP), and postsynaptic spiking leading presynaptic spiking drives long-term depression (t-LTD) [10, 31, 32] (Fig. 1A). This original definition has evolved to include other types of plasticity that depend on spike-timing but are not bi-directional or do not depend on the order of spike coincidence (see [33]). In other cases, postsynaptic spiking leading presynaptic spiking drives t-LTP as in the striatum [34], the adult hippocampus [35] and in the mouse adult primary somatosensory cortex [36]. In addition, numerous basic forms of STDP exist at different synapses with substantial variations, presumably reflecting both synapse specialization and different experimental conditions (reviewed in [8]). Thus, the state of the network (Fig. 1B), neuromodulatory actions (Fig. 1C), developmental stage (Fig. 1D), and astrocytes [37] likely contribute to such diversity [33] and potentially dictate the sign and magnitude of STDP [38].

Timely cooperation, correlated activity, and consequent synchronization of the electrical activity of neurons that are organized in networks also give rise to the emergence of what is known as brain rhythms or neuronal network oscillations. In generalized models, brain rhythms are supported by rhythmic interplay of pyramidal cells connected with inhibitory interneurons within discrete circuits. Inhibitory interneurons play key roles in the rhythm’s generation and maintenance, mainly by efficiently controlling and strengthening the timing of pyramidal cells outputs [4, 17, 39–42]. Rhythmic brain events have been largely documented underlying higher cognitive functions [43–49] and can be recorded macroscopically as an electroencephalogram (EEG) or experimentally/invasively as periodic fluctuations of the Local Field Potential (LFP). Hans Berger reported the first EEG recordings in 1929 [50], describing oscillations below and above 12 Hz as alpha and beta waves, respectively. This practice of classifying brain rhythms by their frequency band is still the current convention, with prominent examples being slow rhythms (<1 Hz), theta oscillations (4–10 Hz), and gamma oscillations (30–90 Hz) [46]. Although the exact frequency range may vary between species, each oscillation band consistently correlates with distinct set of behavioral states and tasks [12, 14, 44, 51].

In local brain circuits, diverse neuronal subtypes are embedded in an organized puzzle of other cellular players such as microglia and astrocytes, which not only modulate but could actively control the concert of information processing. In this regard, astrocytes have been revealed as master regulators of synaptic activity, with diverse functions impacting the computational capacity of synapses and neuronal ensembles [52–59]. This apparent “omniscient” activity is supported by data from e.g., the hippocampus, where one astrocyte covers hippocampal circuits roughly reaching 120,000 synapses [60] established by diverse neuronal cell types. Thus, one astrocyte might be able to detect the neurotransmitters released from those synapses while actively contributing to cooperativity within neuronal ensembles by releasing gliotransmitters [52]. Previous findings together with these and other advances have led to the concept of the “tripartite synapse”, which is composed of a pre-and a postsynaptic neuron with an astrocyte integrated into a functional unit [61]. In turn, microglia, the phagocytic myeloid cells of the brain, have been traditionally regarded as the scavengers of the brain while more recently their contributions to the computational capacity of brain circuits have come into focus [62, 63]. The tripartite synapse concept, therefore, is complemented by microglia, which are particularly attracted by neuronal synaptic activity establishing bi-directional crosstalk [62].

A bi-directional interplay also exists between STDP and neuronal network rhythms where the strength and features of the rhythms are also affected by the occurrence of plastic changes of the synapses involved in the neuronal ensemble [64]. In addition, timing precision could be altered by external inputs with computational consequences, depending on the network architecture and circuit dynamics of the studied structure [38]. Here, we summarize key concepts underlying information processing in the mammalian brain, stressing the crucial role of timing precision for coincident neuronal electrical activity within neuronal circuits. We provide an overview of its consequences on cognitive processes and some behavioral correlates, and we discuss the emerging role of glial cells in STDP, brain rhythms, and their interactions. Technical limitations, controversies and future directions on experimental approaches, and their possible application in humans are outlined.

## Spike timing-dependent plasticity

STDP is a ubiquitous learning rule that has been found experimentally in all species in which it has been studied—from insects to humans [65]. Several forms of STDP involve or require the activation of NMDA-type glutamate receptors (NMDARs) [36, 66–76]. In standard STDP models, postsynaptic NMDARs are suggested to be the exclusive coincidence detector of the spiking activity, and the primary calcium source for STDP [67, 77, 78]. According to the classical view, a presynaptic-before-postsynaptic firing order generates strong NMDAR-mediated calcium signals to drive t-LTP and a postsynaptic-before-presynaptic firing order generates weaker calcium signals that drive t-LTD [79–82]. However, STDP at layer 4-to-layer 2/3 synapses in the primary somatosensory cortex involves separate calcium sources and coincidence detection mechanisms for t-LTP and t-LTD. While t-LTP shows “classical” postsynaptic NMDAR dependence, t-LTD appears independent of postsynaptic NMDARs and instead requires group I metabotropic glutamate receptors and depends on presynaptic NMDARs (pre-NMDARs) [66, 70, 78, 83]. Such unconventional pre-NMDARs are also involved in STDP at other synapse types [76, 84–87], but their function is subject to debate partly due to the technical difficulties in providing unequivocal evidence of their presynaptic signaling to establish a functional link to STDP. In addition, the interactions of pre- and postsynaptic neuronal spiking bi-directionally regulate the intrinsic excitability of pyramidal cells in neural circuits. This has led to the proposition that there is a functional synergy between synaptic and intrinsic plasticity induced by STDP, where LTP and LTD are generally associated with increased or decreased neuronal excitability, respectively (see [88] for review). Finally, there is a great diversity of forms of STDP involving pre- and postsynaptic forms with different underlying mechanisms (e.g., distinct spiking coincidence detection mechanisms [35, 36, 66, 70–72, 78], which also describe different spike-timing windows as shown in Fig. 1. This diversity increases due to neuromodulatory actions [89], differential expression during development [35, 36, 68, 76], and the state of the network or external inputs [38] to the circuit involved, conditioning the order of spike coincidence. In addition, there are diverse forms of STDP which differentially involves specific signaling to and from other cellular players like astrocytes.

### Glial involvement in the control of STDP

Since the emergence of the tripartite synapse concept, mounting evidence has revealed that pre-NMDARs could ally with astrocytes to control information flow in a circuit-specific way, particularly for STDP [90], framing the coincidence as a temporal detection traffic light (e.g., favouring the induction of t-LTD by D-serine release at postnatal days 13–21 (P13–P21), closing a plasticity window due to adenosine release at P22–P30 or further gating a new plasticity window for t-LTP due to glutamate release at P34–P42 in mouse hippocampal CA1 synapses) (Fig. 1D) [35]. These findings and the recent similarities found in the mouse primary somatosensory cortex (S1) [36] allow us to hypothesize that in the mouse hippocampus and S1, astrocytes aid or prevent firing coincidence by providing selective gliotransmitters during coincident spiking depending on the developmental stage. Such dependence on age involves changes of pre-NMDAR expression, glutamate release (by neurons and/or astrocytes), endocannabinoid signalling to astrocytes, timely astrocytic Ca^2+^ signals, and the level of adenosine-mediated inhibition due to astrocytic release of adenosine/ATP in these synapses [35, 36, 66, 68, 76, 91, 92].

Our hypothesis also finds support in three previous unifying hypotheses (see [93] for review) that could explain how astrocytes might influence the spiking coincidence due to the spatial and temporal dynamics of astrocyte Ca^2+^ signals combined with the release of gliotransmitters and actions on neuronal slowly desensitizing and high-affinity receptors. In turn, astrocytes also possess slowly desensitizing and high-affinity receptors for neurotransmitters, which determines the selectivity and the sensitivity of their activation and thus their role on the regulation of synaptic transmission and plasticity. Particularly, the kinetics of astrocyte Ca^2+^ signals (and the subsequent gliotransmitter release) encodes neuronal activity and supports the astrocytic ability to integrate information from different levels of neuronal activity [93] (e.g., rapid and locally restricted Ca^2+^ elevations in response to low synaptic activity modulates synaptic transmission just in the synapse involved, [55] while local Ca^2+^ elevations in response to strong synaptic activity diffuse to nearby processes affecting neighboring synapses [58], or Ca^2+^ elevations at multiple processes in response to coincident activity from multiple synapses drive to an integrated Ca^2+^ elevation, resulting in the control of the synaptic transmission within the whole area occupied by this astrocyte). This decoding and integrating ability provide finely tuned and easily adjustable feedback or feedforward responses that regulate neuronal communication in different time and spatial domains (see [93] for review). In line with this, it is known that neurotransmitter-evoked activation of astrocytes leads to astrocytic release of glutamate, D-serine, ATP, and/or adenosine [94–98], which, through the activation of the corresponding pre- and postsynaptic receptors, establish a threshold for basal synaptic transmission [99, 100] and enhance short- and long-term synaptic plasticity [53, 55, 101]. Astrocytes gradually increase their Ca^2+^ signaling during the induction of t-LTD in a cannabinoid receptor 1 (CB_1_R)-dependent manner at synapses between excitatory neurons in somatosensory cortical layer 4 and layer 2/3 (L4-L2/3 synapses) [97]. Interestingly, at L4-L2/3 synapses of the primary somatosensory cortex, stimulation of astrocytes coincident with afferent activity results in LTD [97]. This has also been observed during coincident stimulation of Schaffer collaterals and astrocytes in the CA1 area of the hippocampus [68, 76].

GABAergic activation of astrocytes also triggers cytosolic Ca^2+^ oscillations and Ca^2+^-mediated signaling in astrocytes [56, 102, 103], which induce a decrease of the excitatory synaptic tone mediated by the release of ATP [104, 105]. Notably, hippocampal astrocytes can decode GABAergic activity based on the frequency and duration of interneuronal spiking activity, thus contributing to neuronal information processing [56, 104]. Such astrocyte-mediated decoding events determine whether astrocytes release either glutamate or ATP and/or adenosine [56], leading to the enhancement or decrease of excitatory synaptic activity and strength, respectively.

It is of particular relevance to note that STDP shows specific developmental profiles in different brain regions [36, 71, 76] and a developmental switch has recently been shown for STDP (from t-LTD to t-LTP) in the CA1 area of the hippocampus [35]. Remarkably, detection of spike coincidence likely changes in this CA1 area with a post-pre protocol, driving a presynaptic form of NMDAR-dependent t-LTD until the 3rd postnatal week in mice [76]. Interestingly, the same protocol induces a presynaptic form of NMDAR-independent t-LTP at more mature stages [35]. As detailed above, astrocytes are involved in this developmental switch by providing D-serine for t-LTD [76] and adenosine and glutamate for t-LTP, and the switch from t-LTD to t-LTP (Fig. 1D) [35]. Recently, a similar switch has been observed in the developing vertical pathways of the primary somatosensory cortex, which intriguingly appears shifted compared to the hippocampus (t-LTD is present until the 4th postnatal week and switches to t-LTP starting at 38 postnatal days [36]). This reveals the need for even more research efforts to understand how plasticity rules, timing, and activity coincidence are differentially governed in diverse brain areas during development and how or whether astrocytes change developmentally. Moreover, although the recognized roles of microglia in neural development, their possible involvement in STDP has so far not been investigated.

Likewise, during postnatal development, microglia play a pivotal role in synapse pruning, aided by fractalkine (CX3CL1) and its receptor. The expression of CX3CL1 increases in the Central Nervous System during embryonic and postnatal maturation, promoting microglial recruitment to neuronal circuits that undergo rewiring during periods of activity-dependent remodeling [106]. In turn, disruption of CX3CL1 signaling leads to cognitive impairment and loss of LTP induced by high-frequency stimulation (HFS-LTP) at hippocampal area CA1 [107]. Interestingly, purinergic receptors located on astrocytes act downstream of microglial-derived ATP to modulate the frequency of excitatory postsynaptic currents (EPSC) in the CA1 area of the hippocampus [108]. In hippocampal area CA3, microglia-derived ATP alters synaptic transmission and short-term plasticity through the activation of presynaptic P2X4 receptors and adenosine receptor 1 (A_1_R), respectively [109]. Additionally, lipopolysaccharides (microglia-selective activators) impair LTP in the rat hippocampal dentate gyrus in vivo. Blockade of A_2A_Rs prevents this by counteracting the shift of microglia toward a pro-inflammatory phenotype [110].

Finally, diverse studies indicate that astrocytes possess mechanisms that allow them to integrate and store synaptic information [94]. Therefore, it has been proposed that astrocytes can “memorize” synaptic events that will have an impact on subsequent neuronal activity. Hence, astrocyte-mediated plasticity is thought of as an activity-dependent and input-specific process that is tightly controlled by synaptic activity. In turn, concomitant neuronal signaling is dynamically modulated by the surrounding astrocytes [52]. Also, it has been observed that myelination (lead by oligodendrocytes) might influence spike timing precision affecting neuronal firing rate, action potential jitter, and latency [26], and this influence may have consequences for STDP. Together, these notions reinforce the concept that brain function relies on interdependent neuron-astrocyte signaling, but the astrocytes’ role in integrating information is yet to be properly elucidated. These studies also indicate that microglia may influence physiological processes such as spiking coincidence and neuronal cooperativity, which may underly aging or complex behavioral patterns related to learning, adaptation, or formation of long-term memory traces.

## Timely coincidence of neuronal activity underlying neuronal network rhythms

**Fig. 2:**
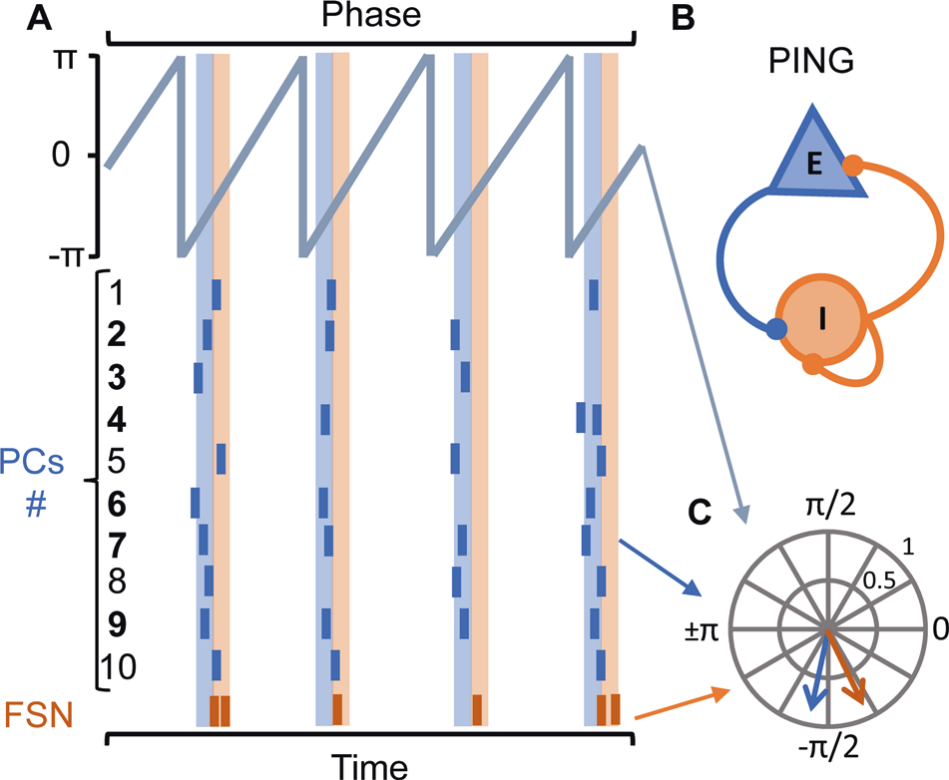
Simplified model of inhibitory fast-spiking interneurons (FSN) and excitatory pyramidal cells (PC) synchronization according to the PING model. **A** Schematic representation of a raster plot showing the action potential firing of ten PCs (blue lines) and one FSN (orange lines) relative to wavelet transform (e.g., Hilbert transform, in gray) of a hypothetical segment of gamma oscillation that emerges from the coordinated activity of represented cells. **B** Simplified circuit showing the synaptic interaction of mutually connected PCs (E: excitatory neuron) and FSN (I: inhibitory interneuron) that give rise to the fluctuations of LFP transformed in (**A**) for instantaneous firing phase detection in the gamma frequency band. **C** Polar plot showing the magnitude (spike-phase coupling) and the preferred phase angle for the hypothetical PCs and neurons shown in (**A**). The phase-lock for PCs and FSN in blue and orange dashed lanes, respectively, can be analytically represented as an infinite circular distribution of neuronal firing according to the instantaneous phase relative to each gamma cycle. A vector length = 1 is assigned to each action potential and the resultant angular summation yield a vector for PC (e.g., PC # 2,3,4,6,7 or 9; blue arrow) or FSN (orange arrow) whose specific angle denotes the preferred gamma phase for firing and the magnitude represents the strength of this preference (phase-lock, synchronization) [122, 195]. This analysis also allows for corroborating that FSN fires after PCs firing and that FSN firing drives a strong inhibition over PCs and entrains them during the gamma cycle in a coordinated rhythmic activity [41, 43, 44].

Brain rhythms emerge from the entrainment of circuits established by direct synaptic contact between neurons and indirect feedforward and feedback connections giving rise to rhythmic electrical fluctuations that can be measured extracellularly [4, 17, 43, 111–113]. These are due, in part, to the spatial and temporal summation of electric current contributions from all active cellular processes within a volume of brain tissue at a given location in the extracellular medium which generate a rhythmic potential [46, 114]. The diverse rhythms of the brain form a hierarchical system that offers a syntactical structure for the spike traffic within and across circuits on multiple time scales [3, 45]. In other words, several interactions occur between brain rhythms (and the neuronal assemblies and intrinsic oscillators generating them), allowing for a hierarchical organization that leads to precise neuronal firing patterns. Another crucial event supporting the generation of brain rhythms is the synchronous firing of neurons involved in the assembly (Fig. 2A). In this regard, sharp-wave ripples (SPW-Rs), which are short-lived ultra-fast oscillations (140–200 Hz) in the CA1 pyramidal cell layer [115], are considered the most synchronous assembly pattern in the mammalian brain [116] and have been early proposed to play a critical role in transferring transient memories from the hippocampus to the neocortex for long-term storage [117, 118].

A possible controversy may arise assuming that the neuronal firing rate instead of the firing synchronization and phase-lock solely underlie the strength and features of brain rhythms. Such current controversy could lie in the experimental configurations, neuronal class recorded, models of study, and/or analytical approaches. For instance, the assumptions needed to analyze spike-LFP phase coupling, the common pitfalls that could bias the analysis (e.g., the firing rate and trials analyzed), and possible ways to avoid these pitfalls based on in vivo studies and computational models have been pointed out [119]. In turn, it has been observed ex vivo that inducing neuronal depolarization with kainic acid (KA) or carbachol leads to persistent gamma oscillations and provides a reliable model to study spike-phase coupling that is not biased by spikes occurrence [43, 120–122]. Thus, it has been observed that rhythmic synchronization and the resultant gamma oscillations parameters (e.g., gamma oscillations power and rhythmicity) are degraded regardless of the firing rate in diverse Alzheimer’s disease (AD) models. This occurs when the firing rate is decreased [122, 123], increased [121, 122, 124], or even when the firing rate remains unchanged [125]. In addition, the increase of spike-phase coupling to gamma oscillations concomitantly with the increase of gamma power and rhythmicity has been observed when the firing rate decreases [120].

Regarding how action potential synchronization in the major neuronal classes contributes to the emergence of brain rhythms, two major models have been proposed. These models focus on explaining the generation and maintenance of the brain rhythm in the gamma frequency band (30–80 Hz; gamma oscillations). Gamma oscillations have emerged as crucial for cognitive processes in the brain and the two mechanistic models attempting to explain their generation are (1) synchronization of the interneuronal networks by mutual GABAergic and gap junction connectivity (the ING model of network entrainment) [126] and (2) synaptic recurrent feedback loops between excitatory pyramidal cells (PC) and inhibitory interneurons (the PING model, Fig. 2B) [43, 127, 128]. Irrespective of the PING or ING model and their respective generalizations, it is established that interneurons play a major role in entraining neuronal circuits. Their spike timing has been extensively studied in diverse experimental models and their unique characteristics have been the subject of several previous experimental works and reviews [4, 17, 39–41, 112, 120, 121, 129–134]. Altogether, these studies suggest that rhythmic synaptic inhibition regulates the spiking activity of neurons and enforces precise cooperativity in local neuronal circuits during gamma oscillations.

In particular, parvalbumin-positive fast-spiking interneurons (PV-FSN) play a pivotal role in the synchronization of PCs during the emergence of the intrinsic hippocampal theta rhythm [17, 131]. This rhythm (theta, 4–12 Hz) has been proposed to support episodic and spatial memory formation by timing the interactions between the prefrontal cortex and hippocampus for memory-guided action selection [135]. In 2015 it was hypothesized that activation of gamma-modulated cell assemblies at a particular theta phase may allow the network to produce a more powerful output by ensuring that cells involved fire closely in time (highly coupled/synchronized). That such a mechanism would serve to facilitate either memory encoding or memory retrieval, depending on which type of gamma rhythm is recruited [136]. Accordingly, targeting PV-FSN entrainment has emerged as a potential therapeutic target to restore normal neuronal network synchronization from impaired, de-synchronized state found in models of neurocognitive disorders such as AD [120, 121, 125, 130, 137, 138]. Conversely, other neurological disorders exhibit hyper-synchronized circuit states or the emergence of “bad” oscillations such as Parkinson’s disease or Epilepsy [139–142]. In these cases, the therapeutic attempts should focus on decreasing the aberrant increase of firing synchronization. Whether these manipulations adapt to, match, or drive a new state of the networks that are intrinsically resonating deserves further research (see [143] for review). Moreover, metabolic arrestment should also be taken cautiously in such interventions, particularly in AD, where energy impairment is a pathology hallmark. How can a system that is undergoing exhaustive metabolic impairment deal with artificially forcing PV-FSN (e.g.) to re-entrain the network into fast rhythmic activity? Notably, PV-FSNs are highly susceptible to energy demands [144] and future studies will consider integrative approaches, including such metabolic arrestment in the generation of synchronous spiking.

In general, brain rhythms at different frequencies can occur separately, but they can also co-exist (nest in each other) and interact. This phenomenon is termed cross-frequency coupling. The most common interactions that have been studied is the coupling between certain features (e.g., phase, amplitude or power) of theta and gamma rhythms [145–149]. This coupling results in a higher frequency (e.g., gamma) rhythm nesting within or on top of the lower frequency (e.g., theta) rhythm at specified phases denoting timing interdependence. Rhythm coupling, particularly theta-gamma coupling, offers physiological advantages such as the possibility of encoding different information in different theta phases and the synchronization of neuronal ensembles over long distances [46, 150]. In addition to the importance of the temporal precision of the rhythm coupling, it has been observed that the theta rhythm not only modulates gamma rhythm but can also induce it [151]. Taken together, these studies reinforce the notion that the timing of neuronal spiking and the timely interaction of brain rhythms is crucial for information processing in discrete circuits and over distant brain areas.

### Glial contribution to brain rhythms

Brain oscillations have classically been considered as purely dependent on the physical and functional contacts between the neurons involved in the ensemble. Non-neuronal contributions have been largely overlooked until recently, but they are starting to be the subject of comprehensive reviews [152]. Glial contribution to oscillations (e.g., gamma) was first described in 2014 [153]. The authors found that blockade of vesicle release in astrocytes attenuates the power and shortens the duration of carbachol-induced gamma oscillations in cultured slices and decreases gamma oscillations power in the mouse neocortex, leading to behavioral changes in a novel object recognition test. Additionally, transient increases in intracellular calcium levels in astrocytes occurs before the onset of carbachol-induced oscillatory activity [153], which might represent an underlying mechanism aiding spike-phase coupling to gamma rhythm. The contribution of astrocytes to brain rhythms has also been observed in vivo [56, 154]. Particularly, specific ablation of GABA_B_ receptors in astrocytes diminishes the power of sensory-evoked theta and low gamma rhythms and impairs theta-gamma phase-amplitude coupling [56]. Furthermore, conditional blockade of gliotransmitter release in astrocytes triggers a critical desynchronization of theta oscillations between the dorsal hippocampus and prefrontal cortex, indicating a modulatory role of astrocytes in the function of widely distant networks [154]. In turn, sensory-evoked gamma oscillations elicit morphological and/or gene and protein expression responses in microglia in diverse mouse models of neurodegeneration, including 5XFAD, APP/PS1, Tau P301S, and CK-p25 mice [130, 155, 156]. One of the hypotheses supporting the effects of gamma entrainment on microglia relies on the high capacity for ion buffering of these cells (e.g., K^+^). This points to the possible direct involvement of these cells in the regulation of ion flow across cortical layers, thus participating in the entrainment [157]. On the contrary, some indirect findings support the notion that microglia activity affects the proper entrainment into gamma oscillations and precise spike timing in the hippocampus [109, 158, 159].

A second hypothesis for the role of microglia in the induction of network rhythms claims a secondary effect instead of a direct involvement in the entrainment [143]. Consequently, microglia could respond to the entrainment since their plasma membranes are exposed to high ionic oscillations and/or are in physical contact with neurons [62, 160, 161]. A third hypothesis considers the involvement of neuromodulatory agents, triggering a secondary effect on microglia due to gamma entrainment. In astrocytes, it has been shown that activation of internal Ca^2+^ levels by several stimuli provokes repetitive NMDAR-mediated responses mainly in CA1 pyramidal neurons. A noticeable feature of this response is that it arises with a high degree of synchrony in multiple neurons through the activation of extrasynaptic NMDARs [59]. An additional aspect to be considered is the emerging role of myelination and, consequently, the role of oligodendrocytes in support of spike timing and synchronization [26]. This highlights the need for more research to dissect the contribution of glial cells (particularly microglia and myelinating oligodendrocytes) to the emergence and/or maintenance of precisely timed neuronal activity within brain circuits during ongoing rhythms.

## Toward an understanding of the complex interplay between STDP and brain rhythms

Does a group of neurons that are firing locked to a specific rhythm-entrained ensemble undergo STDP within the group or with neurons from other rhythm-entrained ensembles at the same time? Do these neurons disengage from the “primary” ensemble to achieve spike timing precision related to STDP, or do neurons undergoing STDP “interrupt” the coincident firing for STDP and start to uniquely fire in a rhythm’s phase-locked manner? These questions represent a technical and intellectual challenge, particularly because of difficulties in determining the ensembles sizes due to their ability to dynamically engage and disengage [3]. However, in a neuronal circuit where neurons are constantly bombarded by multiple external inputs, it is not unreasonable to expect a bi-directional relationship between plasticity and brain rhythms as it is expected in vivo. Due to the heterogeneity of STDP forms, it is challenging to address how STDP directly impacts circuits performance of brain rhythms. Within specific circuits, this interplay could be supported by glial cells due to their key roles in both processes, as previously discussed. For instance, it is reasonable to expect that astrocytes can assist the spiking coincidence and neuronal cooperativity underlying STDP and neuronal network rhythms occurring at the same time within the same ensemble and/or neighboring circuits. This is supported by the astrocytes’ ability to integrate and decode neuronal information occurring in a large array of diverse neuronal activities within complex astrocytes-neuronal interactions due to their previously discussed temporal and spatial dynamics (see [93] for review). Even more sophisticated approaches need to be formulated to test these notions (including simultaneous recording of brain rhythms and Ca^2+^ imaging of astrocytes combined with STDP protocols).

On the other hand, in the hippocampal area CA1, it was found that the timing of tempero-ammonic input relative to theta phase controls the sign of plasticity [38]. Depending on the timing of the stimulation of tempero-ammonic input, it could either advance or delay the postsynaptic spike relative to the theta oscillation, driving a change of outcome: the “disturbance” of the timing without altering the spike firing rate reverses the sign of plasticity and enforces either t-LTP or t-LTD at the Schaffer collateral-CA1 synapse by prospectively controlling postsynaptic spike timing [38]. From this study, we could also assume that, in addition to the presence of external input affecting the sign of STDP, the timing at precise rhythm phases influences the outcome of plasticity, depending on whether the coincidence occurs in the ascending or descending phase of the rhythm. Alternatively, an example of how STDP influences neuronal ensembles performance is that STDP drives synchronous spiking during signal propagation in feedforward networks, which is a common feature in vivo [8]. Also, NMDAR antagonists have been shown to potently increase network hypersynchrony in vivo [162] and it is known that diverse forms of STDP involve NMDARs. As such, affecting them will affect both STDP and brain rhythms occurring at the same circuit.

As mentioned, PV-FSN offer a suitable target to modify the progression of instability in neuronal ensembles that might impact cognition. This has been observed in promising studies in an Alzheimer’s disease mouse model using optogenetic stimulation of PV-FSN at central gamma frequencies [130]. In addition, optogenetic stimulation of glutamatergic neurons from the medial septum and diagonal band of Broca (MS-DBB) strongly synchronizes hippocampal theta rhythms over a wide range of frequencies, probably due to modulation of local septal circuits, which, in turn, contribute to theta rhythms in the hippocampus [163]. The theta rhythms has been proposed to be critical for the temporal coding/decoding of active neuronal ensembles and the modification of synaptic weights. The interplay of theta rhythm and synaptic plasticity in the hippocampus posits the timing of the dendritic excitatory inputs during the theta cycle as crucial for the strengthening and weakening of synapses [113]. In addition, in the hippocampal *stratum radiatum*, the intrinsic oscillatory dynamics of neurons has been found to be plastic owing to the plasticity of voltage-gated ion channel and spatial heterogeneity of pyramidal CA1 neurons. In this region, theta-burst pairing of orthodromic and antidromic stimulations induce t-LTP, and this plasticity is associated with spatially widespread plasticity in resonance and excitability [6]. Recently, it has been observed that selective optogenetic stimulation of CA1 somatostatin-positive interneurons (important players in hippocampal oscillogenesis) restores theta-nested gamma oscillation-induced t-LTP in an in vitro model of AD [137]. Interestingly, in cortical synapses, it has been found that presynaptic spiking produced by synchronous, strong input produces a stronger synaptic response than a spike induced by asynchronous, weak input. This has been named input synchrony-dependent facilitation and involves presynaptic axonal Na^+^ channels that are proposed as “good” readers and transmitters of the levels of input synchrony to the postsynaptic cell [164]. These findings and the proposed model to read and transmit precise levels of input synchrony might have an impact on the circuitry’s computational abilities underlying STDP.

As in many circuits of the brain, strong theta and gamma oscillations coexist in the perforant pathway (PP). In this remarkable input from the entorhinal cortex to the hippocampus, the distal apical dendrites of the basket cell interneurons (BC) preferentially transmit the lower frequencies to the soma [165], thus contributing to the modulation of the PP theta frequency input onto the dentate gyrus (DG). The DG is proposed to be involved in pattern separation and code conversion from neocortical inputs by changing the coincidence patterns and recruiting nonoverlapping cell assemblies in area CA3 [166]. This is possible through the recruitment of feedback inhibition due to the excitation of DG granule cells (GC), which delay the generation of action potentials of less excited GCs [167]. Should repeated activation of GCs in association with BCs occur, their coupling will undergo strengthening through the occurrence of LTP. Consequently, this leads to an improved signal-to-noise ratio maintaining sparse activity in the DG network which is a key requirement for high storage capacity [166]. Then, the progressive emergence of the assembly formed by the highly excited GCs functionally coupled to BCs will plausibly reflect the emergence of a memory trace in the DG network [168]. This integrates plastic events and network entrainment within a feedback circuitry established between excitatory (GCs) and inhibitory (BCs) neurons. Likewise, some forms of synaptic plasticity could modulate the strength of phase-coupled intrinsic and/or extrinsic inputs, revealing an effective mechanism for information encoding. Therefore, STDP could benefit neuronal ensemble engagement and disruption with the leading roles of the participating neurons and the involvement of astrocytes. Contrary, the timing (oscillation phase) of an ongoing rhythm where the spikes coincidence occurs may affect the sign and magnitude of STDP. Moreover, the timing of external inputs relative to ongoing brain rhythms could control the magnitude and sign of STDP although this generalization needs to be further tested in diverse brain areas and synapses.

Together, these findings reveal that there is a finely tuned bi-directional interplay between STDP and neuronal network rhythms that also includes glial contribution. However, rules governing the occurrence of spike coincidence for STDP relative to ongoing brain rhythms and inputs external to the involved synapse are yet to be unveiled. Based on these findings of brain rhythms and STDP interactions, it is tempting to propose that STDP occurring at key synapses of a neuronal ensemble will strengthen or weaken (by t-LTP or t-LTD, respectively) the neuronal contribution and their engagement (spike-phase-locking, rhythmic synaptic transmission) to the rhythm generated within the network. This could impact the computational ability of the involved neuronal network, probably serving homeostatic mechanisms with cognitive consequences [169], as has been observed in computational models [170]. In addition, these findings support the design of experimental protocols mimicking STDP and rhythm interactions [38, 113, 137] and that STDP can modify circuitry properties and vice-versa. Finally, to answer the questions posed at the beginning of this section, detailed studies need to be conducted in a variety of brain circuits by integrating the current knowledge on STDP, brain rhythms, and other external inputs and network states into more comprehensive approaches (e.g., more convergent studies recording brain rhythms combined with STDP protocols are needed).

## Translating insights on STDP and brain rhythms into humans

Translation of the knowledge on STDP and brain rhythms accumulated experimentally in humans is a considerable challenge. Experimental STDP finds support in mathematical modeling and vice-versa, while the physiological evidence of assembly structures driving STDP and rhythms emergence has only arisen recently [8, 38, 132, 171, 172]. A closer correlate in humans for STDP can be found in the transcranial magnetic stimulation (TMS) technique, revealing that STDP-like phenomena exist in humans (see [173] for review). TMS allows for the study of time-locked activation of human cortico-cortical connections in healthy and pathological conditions [174]. It is a non-invasive brain stimulation technique, which uses a time-varying magnetic field to induce electrical currents in cortical areas of interest that ultimately lead to neuronal depolarization and action potential generation [173]. Paired associative stimulation (PAS) is one of the plasticity protocols performed using this technique. It has been shown that repeated pairing of peripheral nerve stimulation with TMS over the contralateral primary motor cortex (M1) area [175, 176] likely operates through STDP mechanisms and is mediated by NMDA receptors [177, 178]. The use of TMS in humans allows for the assessment of cortical function in vivo and can inform us about network characteristics in pathological scenarios such as neurodegenerative diseases.

Brain oscillations have been largely documented in humans aided by the use of EEG recordings [13, 50, 114, 179–182], and are in focus as diagnostic tools, and their manipulation is proposed as a promising therapeutic approach, particularly for AD [130, 162, 179, 182–184]. Accordingly, in neurocognitive disorders, diverse approaches directed at improving or engaging circuits back into functionally relevant neuronal dynamics have recently emerged including optogenetic (experimentally), electrical, or magnetic stimulation [130, 137, 143, 174, 185–190]. The gain-of-function observed in these studies reinforces the notion that STDP and brain oscillations bi-directionally cooperate in humans (as observed in rodent experimental models), and it reveals that by manipulating key players in neuronal dynamics it is possible to promote accurate spike timing in humans.

However, the basal state of the region of interest, the spontaneous occurring rhythms, and their nesting should be further considered in future studies. Whether the induction of de novo formation of neuronal ensembles affects the ongoing rhythms and STDP, its interaction and firing homeostasis demand profound studies of the underlying mechanisms.

## Concluding remarks

Progress has been made towards understanding of how the brain processes information, stores it, and generates reliable and efficien outputs in the form of memories, learning, and other cognitive behaviors. Timing precision is important for brain information processing and such precision is subjected to diverse features and properties at neuronal and circuits levels [191]. As such, intrinsic neuronal resonance and ion channels play important roles in the timing of neuronal spike generation and this timing is plastic. Additionally, within neuronal circuits, spike timing precision leads to STDP and contributes to brain rhythms which are cognitively-relevant. Finally, STDP and brain rhythms interact bi-directionally, and the timing precision necessary for both can be aided by glial cells, whose supportive roles are starting to be uncovered. However, much more remains to be done to elucidate the circuit mechanisms operating to provide functional outputs where timing seems a crucial goal, and comprehensive and careful translation from animal experimental models to humans should be done. Particularly, it appears challenging to compare the oscillations induced by brain stimulation methods with those resulting from currently emerging methods, specially the sensory-evoked circuits’ entrainment [143]. Moreover, the validation of the spike-timing precision underlying STDP and brain rhythms would probably need to be taken (at least for now) from the inherent resulting outcomes of human STDP-like and brain oscillations. In addition, an accurate assessment of the simultaneous occurrence of brain rhythms and STDP in humans (as it may occur) could find a plausible approach in the comprehensive combination of TMS or sensory stimulation and EEG techniques [182, 185, 188, 192, 193].
